# Supplementary material for: Early development of primary care networks in the NHS in England: a qualitative mixed-methods evaluation
Source: BMJ Open. 2021 Dec 28;11(12):e055199. doi: 10.1136/bmjopen-2021-055199 (PMC8719211; doi:10.1136/bmjopen-2021-055199)
Supplement: Author's manuscript [file bmjopen-2021-055199.draft_revisions.pdf]

BMJ Open

BMJ Open is committed to open peer review. As part of this commitment we make the peer review history of every article we publish publicly available.

When an article is published we post the peer reviewers' comments and the authors' responses online. We also post the versions of the paper that were used during peer review. These are the versions that the peer review comments apply to.

The versions of the paper that follow are the versions that were submitted during the peer review process. They are not the versions of record or the final published versions. They should not be cited or distributed as the published version of this manuscript.

BMJ Open is an open access journal and the full, final, typeset and author-corrected version of record of the manuscript is available on our site with no access controls, subscription charges or pay-per-view fees (http://bmjopen.bmj.com).

If you have any questions on BMJ Open's open peer review process please email info.bmjopen@bmj.com

BMJ Open

Holding on while letting go: the early development of primary care networks in the NHS in England

Journal:	*BMJ Open*
Manuscript ID	bmjopen-2021-055199
Article Type:	Original research
Date Submitted by the Author:	05-Jul-2021
Complete List of Authors:	Parkinson, Sarah; RAND Europe, Health and Wellbeing<br>Smith, Judith; University of Birmingham, Health Services Management Centre<br>Sidhu, Manbinder; University of Birmingham, Health Services Management Centre
Keywords:	HEALTH SERVICES ADMINISTRATION & MANAGEMENT, Health policy < HEALTH SERVICES ADMINISTRATION & MANAGEMENT, Organisation of health services < HEALTH SERVICES ADMINISTRATION & MANAGEMENT, PRIMARY CARE

SCHOLARONE™
Manuscripts

I, the Submitting Author has the right to grant and does grant on behalf of all authors of the Work (as defined in the below author licence), an exclusive licence and/or a non-exclusive licence for contributions from authors who are: i) UK Crown employees; ii) where BMJ has agreed a CC-BY licence shall apply, and/or iii) in accordance with the terms applicable for US Federal Government officers or employees acting as part of their official duties; on a worldwide, perpetual, irrevocable, royalty-free basis to BMJ Publishing Group Ltd ("BMJ") its licensees and where the relevant Journal is co-owned by BMJ to the co-owners of the Journal, to publish the Work in this journal and any other BMJ products and to exploit all rights, as set out in our licence.

The Submitting Author accepts and understands that any supply made under these terms is made by BMJ to the Submitting Author unless you are acting as an employee on behalf of your employer or a postgraduate student of an affiliated institution which is paying any applicable article publishing charge ("APC") for Open Access articles. Where the Submitting Author wishes to make the Work available on an Open Access basis (and intends to pay the relevant APC), the terms of reuse of such Open Access shall be governed by a Creative Commons licence – details of these licences and which Creative Commons licence will apply to this Work are set out in our licence referred to above.

Other than as permitted in any relevant BMJ Author's Self Archiving Policies, I confirm this Work has not been accepted for publication elsewhere, is not being considered for publication elsewhere and does not duplicate material already published. I confirm all authors consent to publication of this Work and authorise the granting of this licence.

For peer review only

Holding on while letting go: the early development of primary care networks in the NHS in England

Sarah Parkinson, RAND Europe, Westbrook Centre, Milton Road, Cambridge, CB4 1YG, UK

Judith Smith*, Health Services Management Centre, School of Social Policy, College of Social Sciences, University of Birmingham, 40 Edgbaston Park Rd, Birmingham, B15 2RT, UK

Manbinder Sidhu, Health Services Management Centre, School of Social Policy, College of Social Sciences, University of Birmingham, 40 Edgbaston Park Rd, Birmingham, B15 2RT, UK

***Corresponding Author,** Health Services Management Centre, School of Social Policy, College of Social Sciences, University of Birmingham, 40 Edgbaston Park Rd, Birmingham, B15 2RT, UK.

Email: j.a.smith.20@bham.ac.uk Tel: +44(0)121 414 7068

Word count excluding title page, abstract, references figures and tables: 5001

Abstract

Objectives: Primary care networks (PCNs) were introduced in the NHS in England in 2019 to improve integrated care for patients and help address financial and workforce sustainability issues in general practice. The purpose of this study was to collect early evidence on their implementation and development, including motivations to participate and what enables or inhibits progress. This paper considers the core characteristics of PCNs, and how this informs their management.

Design: A qualitative mixed-methods rapid evaluation was conducted across four case study sites in England, informed by a literature review and stakeholder workshop. Data collection comprised interviews, non-participant observation of meetings, an online survey and documentary review.

Results: General practitioners (GPs) are motivated to participate in PCNs for their potential to improve patient care, enable better coordinated services and enhance financial and workforce sustainability within primary care. However, PCNs also have an almost mandatory feel, based on the national policy context and significant financial incentives associated with joining them. PCNs offer potential to bring general practices together to work towards common goals, deliver national priorities and respond rapidly to local needs.

Conclusions: PCNs face similar challenges to other meso-level primary care organisations internationally, as they respond to local and national priorities and operate in a context of multiple goals and interests. In managing these organisations, it is important to find a balance between local and national autonomy, decision making and control.

For peer review only - http://bmjopen.bmj.com/site/about/guidelines.xhtml

Strengths and limitations of this study

- As a rapid evaluation, this study responds to current policy-relevant questions about the early development of PCNs, and developments in how primary care is delivered in the NHS in England.
- The qualitative approach provides insights into why GPs participate in PCNs and the experience of implementation.
- The mixed-methods approach to this evaluation allows data to be triangulated between sources and ensures that a broad range of perspectives is captured.
- The use of a theoretical framework to interpret the findings from this evaluation helps contextualise them within the wider literature, and understand what this evaluation means for other meso-level primary care organisations internationally.
- This evaluation provides an insight into the early development and implementation of PCNs, along with information about their initial response to the COVID-19 pandemic. Although the study reflects on how PCNs will continue to develop, for example in response to new policies in the English NHS, definitive conclusions about the impact of PCNs were outside the scope of this study.

Conflict of interest

No conflicts of interest are declared.

Ethical approval

An application for ethical review to the University of Birmingham's Research Ethics Committee was made by the project team and approval was gained in July 2019 (ERN_13-1085AP34).

Funding sources

This project was funded by part of a grant from The National Institute for Health Research, Health Services and Delivery Research programme (HSDR 16/138/31 – Birmingham, RAND and Cambridge Evaluation Centre).

Acknowledgements

We thank Amelia Harshfield, who contributed to the conception and design of the study, and data collection at two study sites. We also thank Natasha Elmore, Dr Sarah Ball, and Jon Sussex (all from RAND Europe) for their contribution to data collection, gaining ethical approval, and providing project advice; Dr Rebecca Fisher (Senior Policy Fellow, Health Foundation) and Dr Mina Gupta (GP, Modality Partnership) for peer review of our online surveys; Samantha Hinks (NHS England and Improvement) and Professor Katherine Checkland (University of Manchester) for their on-going advice and sharing learning from their own evaluations and research throughout; Professor Russell Mannion (University of Birmingham), Dr Katie Coleman and Mark Platt (BRACE Centre Health and Care Panel) who reviewed our study protocol; and Professor Justin Waring (University of

For peer review only

Birmingham) and Dr Anna Dixon (formerly Chief Executive, Centre for Ageing Better) for undertaking critical review of our findings.

Author contributions

JS was principal investigator for the study and responsible for its conception, design, conduct and writing up, with support from MS and SP. MS, SP, and JS undertook data collection and SP led data analysis supported by JS and MS. SP wrote the first draft of this paper, and MS and JS commented on and contributed to drafts.

For peer review only

INTRODUCTION

Primary care networks (PCNs) are the latest in a long line of general practice (GP) collaborations in the NHS in England dating back to GP fundholding and locality commissioning in the 1990s.[1] Predecessor collaborations have encompassed a wide range of arrangements, including total purchasing projects, primary care groups and trusts, practice-based commissioning, personal medical services schemes, and clinical commissioning groups.[2-4] These collaborations have ranged from informal networks to formal multi-site practice organisations and super-partnerships where GP practices merge important functions such as managing finances and contracts.[5] These other forms of collaborations have had varied aims, including improving care at a local level and delivering new services to patients, strengthening the resilience of general practice, and supporting better management in primary care, including improved financial stability.[2, 6]

PCNs were introduced in 2019 as part of the NHS Long Term Plan,[7] which claimed that these new networks would create integrated and community-based healthcare, support expanded neighbourhood teams, increase workforce sustainability and deliver on a number of national priorities such as health inequalities and early cancer diagnosis. The NHS Long Term Plan announced that at least £4.5 billion would be invested in these networks over the following five years. Since this time, nearly all practices in England have joined a PCN.[8] PCNs were introduced into the English NHS at a time of particular financial and workforce sustainability challenges in primary care and general practice,[9-11] which is important in understanding their goals and policy context. Key characteristics of PCNs are set out in Box 1 below.

One notable way in which PCNs depart from some previous forms of collaborative working is that many prior collaborations (for example, GP super-partnerships, GP federations, GP multifunds) evolved from the ground-up, meaning that local actors within primary care had taken the initiative to work together out of necessity or shared interest. In contrast, PCNs have been encouraged through national policy with significant financial incentives,[12] giving them a compulsory, top-down feel when compared to some previous forms of collaborative working, although they share this more mandated approach with primary care groups, primary care trusts, and practice-based commissioning.[13] While participation in PCNs is in theory voluntary for GP practices, in reality almost all practices have interpreted them as mandatory, considering the significant levels of new funding that are distributed through PCNs.[14]

For peer review only

Box 1: Key features of PCNs

- PCNs are intended to bring together groups of neighbouring (geographically contiguous) GP practices, along with other primary care providers such as community pharmacies, dentists, optometrists and voluntary sector organisations.
- The NHS Long Term Plan specified that PCNs should cover a patient population of 30,000 to 50,000 patients,[7] although many now cover much larger populations (upwards of 100,000 to 150,000 patients)[15]
- PCNs receive funding on a per-patient basis for enhanced services and additional funding to support recruiting new shared roles such as social prescribers and clinical pharmacists. This funding is distributed through their local clinical commissioning group (CCG, the local funding agency which commissions most hospital, mental health and community services in local areas in England).
- Each PCN is led by a clinical director, who receives funding for their role depending on population size of the network, weighted by deprivation and burden of morbidity.[14]
- The Direct Enhanced Services (DES) contract[16] specifies what services PCNs must provide to gain access to funding, and includes specifications for structured medication reviews, general practice support of care homes, anticipatory care for patients in the community with complex needs, early cancer diagnosis services, cardiovascular health, and health inequalities. The contract sets out what local commissioners of primary care services must offer to providers participating in PCNs, and what services PCNs must deliver to receive additional funding.

Primary care networks are meso-level organisations,[6] operating between formal funders or commissioners, and local GP practices. As such, they are somewhat hybrid in nature, being both national and local, and extrinsically (e.g. based on policy and incentives) and intrinsically motivated (e.g. based on expected benefits and desire to collaborate) through a national policy initiative as well as shared goals and interests. As meso-level organisations, PCNs share characteristics with international experiences of primary care organisations, displaying complexity in their form, objectives and ways of working,[14] and occupying a sometimes unclear position within national and local healthcare systems.[17]

As networks of healthcare professionals, there is also much to learn about PCNs from prior work on the characteristics of professionally-led networks and healthcare network management. The existing literature explores effective ways to manage and govern networks in healthcare, depending on the structure of the network and the context within which the network is functioning.[18-22] This paper contributes to this body of literature, and applies existing theoretical work on healthcare networks management to the early experience of PCNs in the NHS in England.

This analysis draws on a rapid mixed methods evaluation of the first year of operation of PCNs[13] to explore their implementation and early progress. The findings are interpreted using theory about the nature of health care network structure and management, drawn from work by Goodwin et al. (2004).[23] In particular, this analysis includes an examination of the characteristics that PCNs share with 'enclave networks', with a rather flat organisational structure, formed of relatively close-knit groups of professionals and seeking to have a bottom-up and locally-owned sense of purpose, as well as 'hierarchical networks,' designed to undertake specific tasks as dictated through contractual and funding mechanisms that are enacted in a top-down manner on behalf of a national health system.[23]

This analysis addresses the following questions:

review only

- RQ1: What was the rationale for GP practices to join and participate in a PCN?
- RQ2: What enabled or inhibited the early progress made by PCNs?
- RQ3: What are the core characteristics of PCNs, given their role as meso-level organisations working between local general practice and national health funders and commissioners?
- RQ4: What does this experience reveal about how to manage and prepare meso-level primary care collaborations to fulfil local and national policy expectations?

METHODS

The rapid evaluation study explores four case study sites across England through documentary review, interviews, a survey, and non-participant observations, which are described below. The full findings from this research are described in Smith, et al. (2020),[13] while this article draws on additional analysis of evaluation data to address the research questions set out above, and as detailed in Table 1.

Table 1: Summary of methods and research questions

Study phase	Description	Research questions
Rapid evidence assessment	An overview of published evidence to distil prior learning and inform the development of propositions to be tested through comparative case studies of new primary care networks	RQ1 and RQ2
Stakeholder workshop	A workshop led by members of the study team for relevant stakeholders (e.g. academic and policy experts in the field, patient and public involvement representatives).<br>The aim of this workshop was to clarify evidence gaps and evaluation questions of particular relevance to emerging policy on primary care networks and thus inform next steps.	RQ1 and RQ2
Comparative case studies of four primary care networks	Interviews with those involved in the conceptual design, implementation of primary care networks in their respective sites, and exploration of relationship with any prior GP collaboration in the case study site; analysis of key documentation (both internal and publicly shared); non-participant observation of strategic meetings; and an	RQ1, RQ2, RQ3 and RQ4

	online survey to collate information on challenges associated with collaborative working and measuring early impacts.	
Analysis of findings from case studies to develop a nuanced understanding of the development of primary care networks in the NHS in England	Share and discuss findings generated from data collection from case studies.	RQ1, RQ2, RQ3 and RQ4

Patient and Public Involvement

A half-day project design workshop was undertaken in November 2018 and involved, in addition to the research team, national primary care policy officials, a patient representative, academics with experience of researching primary care organisations, and policy experts in the field (N=12). The aim of the workshop was to help identify gaps in the literature, and thereby devise relevant research questions. Participants at the workshop felt a key unexplored area was the experiences of primary care collaborations in rural, as opposed to urban areas, to better understand regionally-specific challenges in primary care. Furthermore, attendees were keen for researchers to investigate sites where it had proved challenging to sustain primary care collaborations, and to examine what management and organisational development skills and capacity are needed to make a PCN work effectively.

Sampling and recruitment of case study sites

Purposive sampling was used to select sites that had not been involved in research studies or evaluations over the previous two years, and to ensure that the sample included rural sites, as well as collaborations that had previously faced challenges in sustaining collaborative working. Potential clinical commissioning groups (CCGs) to approach were identified using a combination of an online search of grey literature and those that had responded to a 2017 study by the Royal College for General Practitioners.[24] Twenty-eight CCGs were contacted as potential participants from May to August 2019, and those that responded (n=7) were sent a short survey to determine whether emerging PCNs in their area met the inclusion criteria. Three case study sites were identified using this approach, and a fourth was identified through engagement with providers known to the researchers. Three case study sites were PCNs, and one was a super-partnership with member practices also belonging to several PCNs. A short description of the four case study sites is provided in Table 2 below and a summary of the sampling approach is illustrated in **Error! Reference source not found.**

For peer review only

Table 2: Description of case study sites

Case study site	Short description
Site A	PCN in a rural setting covering a patient population of 75,000 (large ageing population, mostly White British), where practices had previously worked together through an informal model of locality working. Some practices in the PCN were also involved in a super-partnership.
Site B	Super-partnership in a rural setting covering a patient population of 130,000 patients (large ageing population, mostly White British). Practices within super-partnership were part of four separate PCNs which also contained non-super-partnership practices.
Site C	PCN in an urban and semi-urban setting, covering a patient population of about 60-70,000 patients (socioeconomically disadvantaged population, significant Black, Asian and Minority Ethnic population), where practices had previously worked together formally in a GP Neighbourhood.
Site D	PCN in a rural setting, covering a population of 30,000 patients (large ageing population, mostly White British), where practices previously worked together and with community teams informally.

Data collection and recruitment

Data collection was facilitated through a gatekeeper,[25] or contact point, at each case study site. A total of 29 semi-structured interviews with 25 participants were conducted using a topic guide (summarised in Box 2), each lasting between 30-60 minutes. Participant characteristics are described in Table 3. A minimum of one and a maximum of nine interviews were conducted at each of the four case study sites with both clinical and non-clinical staff, mainly with those in leadership or management positions within the PCN. Interviews were audio recorded, transcribed verbatim using a professional transcription service and pseudonymised. Four of these interviews were follow-up interviews with PCN managers to gather information on their response to Covid-19. Interviews were completed both face-to-face and virtually (due to the onset of the Covid-19 pandemic) by JS, SP, MS and two further researchers with qualitative interviewing experience. Data saturation was achieved for themes regarding rationale for GP practices to join and participate in a PCN and what may have inhibited or enabled progress; although saturation may not have been achieved for themes focused on the trajectory of primary care networks in the post-pandemic English NHS given that data collection ended in the initial phase of the pandemic.

Nine meetings (e.g. board- or partner-level meetings, task group meetings) were observed across the four case study sites by SP, MS and two other researchers with experience in non-participant observations. A template was used to take notes at each meeting on the topics discussed and dynamics within each case study site, including a sociogram to visualise how meeting participants interacted with one another.[26] For both interviews and observation, participants were given an information sheet about the study, given the opportunity to ask questions, and provided informed consent prior to data collection. Lastly, gatekeepers provided access to key documents at each site, including material related to the structure of the PCN and any pre-existing GP collaboration, governance and decision-making, agendas of previous meetings and local communication activities. Information was extracted from these documents using a structured Excel template based on the aims of the evaluation.

For peer review only

Box 2: Interview topics

1. Models of GP collaboration in the local area and how previous and extant collaborations relate to PCNs
2. Specific challenges to PCN working, particularly in relation to urban and rural settings, and any practice that may have left PCNs
3. How collaborative working in local primary care systems has evolved since introducing PCNs
4. Nature of professional relationships within PCN
5. Motivations to participate in PCNs
6. Key goals and outcomes for short and medium to long term for PCNs
7. Early impacts of PCNs

Table 3: Characteristics of interviewees from four case study sites

**Site**	**Description**	**Number (N)**
Site A	Primary care clinical staff	4 (Int1-4)
	Primary care non-clinical staff	5 (Int5-9)
Site B	Primary care clinical staff	3 (Int10-12)
	Primary care non-clinical staff	3 (Int13-15)
	Clinical commissioning group staff	2 (Int16-17)
Site C	Primary care clinical staff	4 (Int18, 20-22)
	Primary care non-clinical staff	2 (Int23-24)
	Clinical commissioning group staff	1 (Int19)
Site D	Primary care non-clinical staff	1 (Int25)
**Total**		**25**

Synthesis and analysis

After data had been collected, the evaluation team (JS, SP and MS) participated in a half-day data analysis workshop to review data collected, discuss themes and begin systematic analysis of the data

For peer review only

as per the framework method for data analysis described in Gale et al. (2013).[27] Data from interviews were analysed through deductive coding with NVivo 12 software[28] using a codebook that had been developed by the evaluation team based on the evaluation aims, available literature on primary care collaborations and initial reading of interview transcripts. Analysis was led by SP, whereby an initial coding frame was developed based on codes arising from a sample of five transcripts by MS and SP. MS and SP coded all transcripts, and further developed codes based on subsequent transcripts and further discussions. This approach was also applied to data from non-participant observation meeting notes and documentary review template. After analysis, themes were discussed in a second half-day workshop (JS, SP, and MS) with the evaluation team to synthesise evidence for each of the research questions and develop an overarching narrative summary (written by JS) of the findings.

FINDINGS

Analysis of data from evaluation fieldwork highlights the rationale for general practices to join PCNs, what has facilitated and inhibited the early progress of these new networks, and what this means about the nature of how PCNs operate and are likely to develop longer term. The full findings of the evaluation are reported elsewhere,[13] while this secondary analysis of data from the evaluation focuses on interpreting the data in relation to health care network structure and management.

Reasons for joining and participating in a PCN

There are many reasons why GP practices join and participate in PCNs, these being based on both top-down and bottom-up motivations. These reasons reflect the policy and incentive structure that led to the introduction of local PCNs within the national context of the English NHS, as well as a genuine desire to collaborate locally to ensure the sustainability of primary care and improve and enhance the services available to patients.

When asked about the reasons why their practice joined a PCN, interviewees involved in practice-level management reflected that PCNs are, in effect, perceived to be mandatory given the sizeable financial incentives associated with PCN membership. There was some sense of frustration about the perception that practices have been forced or coerced into joining PCNs, although others asserted that the national PCNs policy is based on the known efficacy of primary care in responding to incentives.

> *'Most my GP colleagues in other practices and within my partnership, we all were very suspicious of it and also didn't feel it was the right mechanism for delivering the resilience in general practice which we need because it was being foisted on us… it was the only way we could see that we were going to get any new money coming into general practice…. I guess we thought… we might as well.'* (Int1)

Despite this focus on top-down motivations, bottom-up motivations also contributed to the desire to join a PCN. All four case study sites had a history of their general practices collaborating with one another to some extent, either through informal groupings or more formal arrangements such as super-partnerships or locality forums. Respondents involved in the management of PCNs reflected that practices typically collaborate to fill gaps in the services that single practices are able to provide, and to facilitate GPs working with community health teams, social services and the voluntary sector to provide extended care that addresses local population health needs.

> *'We're only a small network, 35,000 patients in the network… I sort of see the 35,000 rather than the 3,000 we've got on our list. So I'm really enthusiastic, and I want to make sure that the 35,000 are looked after, as much as my 3,000'*

For peer review only

A clear desire to improve the sustainability of primary care also was a shared goal that motivate GP practices to work with one another within PCNs. Some interviewees also mentioned that working in collaboration across practices is attractive because of the potential for financial efficiency and sustainability by sharing back office functions, reducing duplication of administrative tasks, introducing more robust financial management processes and making it easier to recruit and retain new staff, for example, by providing more opportunities for training, education and specialisation.

The reasons for joining and participating in PCNs impact not only on individual GP practices, but also the structure of PCNs themselves. Networks can be built on the shared interests, goals and motivations of members, and also through formalised structures and top-down regulation that require or incentivise membership. In the case of PCNs, members are bonded by a blend of these structural mechanisms. National policy has prompted the forming of PCNs, but in the absence of national policy incentives, it would remain in GP practices best interests to still collaborate with one another to provide services, improve management and realise efficiencies based on their mutual interests. This blend of motivations influences the relationship that network members have with one another, and also the place of the network within the wider health and care system.

Local engagement and ownership of PCNs

Engagement by practices in the PCN at a local level is critical to ensuring that networks not only deliver the national priorities set for them, but also address local health needs and improve the integration of services across primary care. This is of particular importance given NHS policy direction towards new integrated care systems.[29]

Early in the implementation of PCNs, there tended to be little engagement with the PCN below the leadership and management level among staff in constituent GP practices. At this stage, there seemed to be a sense that PCNs had not yet had much effect, and that local practices would continue to deliver services for patients and operate much as they did before PCNs.

> *'Some of the staff wouldn't know that we were in a network, even though we've told them about it. If you then said about the PCN, they'd say well what's that?'* (Int6)

In some cases, this lack of engagement was reported to be exacerbated by a perception that PCNs were the latest in a long line of collaborative mechanisms set out by the NHS for GPs. Frustration was expressed about frequently changing NHS policy that disrupts extant ways of working, including activity under way to improve patient care through other forms of locally developed primary care collaborations such as federations and super-partnerships. There was also some irritation expressed by interviewees and observed in meetings around the prescriptive nature of the services required by the DES contract,[16] which further tempers local buy-in to PCNs, particularly where services specified in the DES contract are perceived as not tailored to the needs and preferences of local populations.

> *'We just thought, well we've been there before. We deal with the box ticking. Get the box ticking done and then deliver what… might improve care for our patients'* (Int1)

There was also genuine enthusiasm expressed by some interviewees for PCNs as a sign of greater investment in NHS primary care, and as a way to raise the collective voice of GPs and primary care, for example in terms of negotiating collective contracts. Some of those involved in the leadership and management of PCNs expressed that they have experienced a sense of empowerment in working on something larger than a single practice, and being involved in strategic planning of local primary and community health services over and above single-practice working.

For peer review only

'The main thing that has come in – and this isn't just here – is the enthusiasm with which mostly a new set of GP faces have really taken on a new role and are invigorated and believe they're a bit empowered, and they're doing something at a bigger, more strategic level than out of practice' (Int19)

Where PCNs are perceived as a continuation of existing efforts to improve general practice sustainability and local healthcare, there seems to be a high level of enthusiasm and buy-in. However, where PCNs are perceived as a disruption to previous ways of working and a divergence from the goals of pre-existing forms of GP collaboration, there seem to be tensions and frustrations. On balance, engagement and buy-in will need to be fostered in order to build support for PCNs among wider primary care teams, and to ensure that those involved in managing and leading PCNs remain dedicated to their success.

The level of local engagement with and ownership of PCNs is connected to how they are structured as networks. Where PCNs are felt locally to be part of existing efforts to improve care, population health management and practice management, more individuals within GP practices appear to have bought into the premise of network working. In turn, the network is perceived to be founded upon shared goals and interests, and less on top-down mechanisms that contractually bind network members together. However, the opposite is also true. Where PCNs are thought to be another top-down policy change, fewer individuals buy into the idea of primary care networks, and there is likely to be increasing frustration about top-down interruptions to existing ways of working at a local level.

The role of PCNs in the local health system

This evaluation explored the first year of the development and implementation of PCNs, as they were still finding their place within the wider health and social care system. Different local contexts, for example relationships with statutory NHS bodies and histories of previous collaborative working, contributed to a diversity of ways in which PCNs have been working within local healthcare systems. As the COVID-19 pandemic emerged in 2020, this also influenced the role of PCNs within the local and wider NHS.

One way in which this variation played out was through the relationship between PCNs and local clinical commissioning groups (CCGs, which commission most hospital and community services in local areas in England). Some PCNs had drawn on management support from the local CCG throughout their development and implementation, while other PCNs reported little involvement from the CCG, or even cases of tension where the CCG was perceived as exerting undue influence over PCN priorities and budgets.

Variation in local context was also evident in the relationship between PCNs and pre-existing forms of GP collaboration, including GP federations and super-partnerships. At times, PCNs had been able to build on good working relationships established from previous collaborative working between practices and with other parts of the health and social care system and voluntary sector, which helped establish the position of PCNs locally. In one case study site, the super-partnership exerted considerable influence on PCNs to which member practices belonged, to the extent that PCNs merged and expanded to fit the geographical boundary of the super-partnership. These shifts will inevitably affect an individual PCN's place within the local health and care system and the scale at which the PCN operates in terms of its patient population.

Lastly, the COVID-19 pandemic has further shaped the place of PCNs within local and national health and social care systems.[30] PCNs have been an important mechanism in delivering the national

COVID-19 vaccination programme and have led the designation and deployment of vaccination sites after being asked to do so by NHS England and Improvement in December 2020.[31] Locally, PCNs were key to organising the delivery of primary care during the pandemic, for example by organising 'hot' and 'cold' hubs to care for COVID-19 and non-COVID-19 patients, and helping to coordinate the movement of staff between practices.[13] PCNs' role in both national and local healthcare delivery during the pandemic has already influenced their role within the health and social care system (e.g. by influencing national priorities that PCNs will focus on, including long COVID and weight management[32]), in ways that will likely become clearer as England emerges from the pandemic.

The place of PCNs within the wider health and care system is also linked to how they are structured and gain legitimacy as networks. Depending on the PCN's relationship with other organisations locally and nationally (e.g. with CCGs and local super-partnerships), and the demands being placed on PCNs due to system-level pressures (e.g. the pandemic), the place of the PCN within the wider system shifts. At times, the PCN is a mechanism for collaboration on certain, specified tasks, while at other times, it is a primary unit to deliver critical tasks such as primary care's pandemic response, and a focal point for interaction between local primary care and wider systems.

DISCUSSION

This evaluation reveals that PCNs, while introduced through national policy, are also based on shared goals of improving sustainability in primary care and improving integrated services for patients. While they are organised around delivering a set of priorities set out in the national DES contract,[16] they are also firmly based in local health and care systems, dependent on their local context and population health needs. Beyond their initial development and implementation, a challenge for PCN will be to build buy-in and engagement and clarify their place within the wider health and care system. To support PCNs as they continue to develop, and to ensure they are able to address both national priorities and local health population needs, including health inequalities, it will be important to ensure that appropriate management structures are in place, while also giving PCNs sufficient autonomy to adapt.

Although PCNs specifically are unique to the English NHS, thinking about what support they are likely to need to address local and national priorities longer term is informative for wider discussion of the international experience of meso-level primary care organisations. Primary care organisations in other jurisdictions find themselves, like PCNs, shifting between a focus on local and national health priorities, and face challenges finding their place in wider health and care systems. They also report the common risk of being swept into increasingly centralised functions such as those identified in national policy initiatives.[17]

Goodwin et al. (2004)[23] provide a lens for thinking about the kind of management and support that PCNs and similar international examples of primary care organisation may need to ensure that they can reach their full potential. The authors establish a typology of three types of networks, based on the level of social regulation and social integration within the network (see Table 4).

Table 4: Different networks structures – Adopted from NHS SDO (2004),[33] based on Goodwin et al. (2004)[23]

Network type	Key characteristics	Key lessons for network management

For peer review only

**Enclave**	• High social regulation and low social integration<br>• Equality between members, flat internal structure<br>• High level of social cohesion and share commitment to common interests, values and goals	• Creates bottom-up legitimacy and promotes creation of new ways of working<br>• May fail when motivation of members is exhausted or when tensions occur<br>• Management may be administrative, helping to facilitate collaborative working, but without formal audits
**Hierarchical**	• High social regulation and high social integration<br>• Centred around organisational core that is able to regulate its members<br>• May be sustained by common interests, values and goals, but also based on structured agreements and protocols	• Most successful in coordinating and executing pre-defined tasks<br>• May fail through over-regulation, which limits ability to innovate and leads to low motivation of members<br>• Management to coordinate defined activities and provide central direction, although it is suggested that mandated networks should be avoided
**Individualistic**	• Low social regulation and low social integration<br>• Single entities or organisations that come together to achieve certain tasks<br>• No strong sense of shared interests, values and goals	• Innovative and flexible, with fluid membership<br>• May fail due to high cost of membership, competition and conflict between members that can limit desire to work jointly<br>• Management may help set targets, incentives and monitoring activities

PCNs can be understood both as enclave and hierarchical networks. They are simultaneously founded on shared goals and motivations and a relatively flat structure whereby each practice within the PCN has a voice, as well as being organised to be able to execute pre-defined tasks specified in the DES contract based on the national policy and funding infrastructure that initiated and surrounds them. Examining PCNs through this theoretical lens allows a more nuanced approach to the support that PCNs will require going forward, including in addressing the issues that PCNs face in terms of securing local ownership and engagement, and clarifying their role within the wider health and social care system.

As enclave networks, PCNs share the common goal of wanting to ensure sustainability in primary care, including financial and workforce sustainability, and improving integrated services that meet the needs of the patients of constituent practices. Locally, there is a preference for focusing on the characteristics that PCNs share with enclave networks, as evidenced by the enthusiasm and commitment that was expressed for the underlying goals of PCNs and the ability to work collaboratively to address local population health needs, as compared to the reticence and frustration towards the top-down, prescriptive nature of PCN policy, particularly where they were not perceived to be aligned with local priorities. Fostering this sense of shared goals and intrinsic motivation may help encourage buy-in and engagement with PCNs, and allow them the space and autonomy to arrive

For peer review only

at solutions that address local population health needs. Even as PCNs continue to address national health priorities and complete pre-defined tasks, it will be important to balance and align these with local priorities to foster buy-in, engagement and a shared sense of interests and goals within PCNs.

PCNs also share characteristics with hierarchical networks – they emerged from a centrally-determined policy and funding mechanism, and are designed to deliver services as set out in the national specification for PCNs.[16] In this sense, PCNs are well-suited to deliver on pre-determined tasks and respond to direction and guidance from central bodies, and have been effective in quickly making progress towards national strategic goals by establishing new and enhanced services for patients. However, as hierarchical networks they face a risk of over-regulation and excessive performance management that could inhibit motivation and enthusiasm for PCN teams and hamper their ability to innovate locally, which has been an issue for predecessor primary care organisations in the past.[34, 35]

The risk of over-regulation will be especially important to consider as the proposed integrated care systems (ICSs) are implemented nationally, CCGs are abolished, and PCNs likely find themselves having to work out their role within a restructured NHS.[36] PCNs have been identified as critical to the future success of ICSs by NHS England and Improvement and the Department of Health and Social Care,[29] which will likely have implications in terms of how PCNs are organised. It is possible that PCNs will come under pressure to grow in size and complexity, merge with neighbouring PCNs, which will add to the challenges they face in terms of local engagement if these risks are not carefully mitigated. The risk that PCNs are increasingly drawn into formal hierarchical arrangements and mergers is a common experience among meso-level organisations in primary care in the international context.[37-39]

CONCLUSION

This evaluation reveals that PCNs demonstrate significant potential to swiftly deliver new services to patients, respond to national priorities, bring together primary care providers with common motivations and interests, and improve financial and workforce sustainability in primary care. Furthermore, during the pandemic PCNs have responded to both national priorities in their participation in England's vaccination programme, as well as rapidly responding to local needs, for example by coordinating the movement of staff and patients between 'hot' and 'cold' hubs.

The task ahead for PCNs will be to ensure that they are able to address national priorities that are centrally defined, as well as adapting to fit local health needs. Focusing on the shared goals that motivate GP practices to want to collaborate with one another, and protecting PCNs from over-regulation, will be especially important as PCNs find their place within the wider NHS as it emerges from the pandemic, and as integrated care systems (ICSs) are implemented.

Primary care organisations like PCNs are often strongly placed to address local and national needs, being both enclave and hierarchical in nature, and should continue to address both of these areas. Careful attention needs to be paid to how these priorities are balanced, and how decisions are made that shape how these organisations fit into wider health and care systems. In order to enable these organisations to reach their full potential, the core characteristics of these organisations must be considered in deciding how they should be managed, including the motivations why individual providers join these organisations and the policy context that led to their development.

For peer review only

REFERENCES

1. Smith J, Harshfield A. Primary care networks: a marathon not a sprint. *Health Services Journal* 2019.
2. Pettigrew L, Nicholas Mays, Stephanie Kumpunen, Rebecca Rosen. *Large-scale general practice in England: what can we learn from the literature?* Nuffield Trust; 2016.
3. Exworthy M, Mannion R, Powell M. *Dismantling the NHS?: Evaluating the impact of health reforms*; 2016.
4. Alderwick H, Dunn P, McKenna H, Walsh N, Ham C. *Sustainability and transformation plans in the NHS: how are they being developed in practice?* London: The King's Fund; 2016.
5. Rosen R, Stephanie Kumpunen, Natasha Curry, Alisha Davies, Luisa Pettigrew, Lucia Kossarva. *Is bigger better? Lessons for large-scale general practice*. Nuffield Trust: ISBN: 978-1-910953-12-9; 2016.
6. Smith J, Goodwin N. *Towards managed primary care: the role and experience of primary care organizations*: Ashgate Publishing, Ltd.; 2006.
7. NHS England. *The NHS Long Term Plan*; 2019.
8. Baird B. *Primary care networks explained*. London: The King's Fund; 2019.
9. Charlesworth A, Gershlick B, Firth Z, Kraindler J, Watt T. *Investing in The NHS long term plan: Job done?* London: The Health Foundation; 2019.
10. Fisher R, Turton C, Gershlick B, Alderwick H, Thorlby R. *Feeling the strain: What The Commonwealth Fund's 2019 international survey of general practitioners means for the UK*. London: The Health Foundation; 2020.
11. Nelson P, Martindale A-M, McBride A, Checkland K, Hodgson D. Skill-mix change and the general practice workforce challenge. *Br J Gen Pract* 2018;**68**:66. https://doi.org/10.3399/bjgp18X694469
12. Fisher R, Thorlby R, Alderwick H. *Briefing: Understanding primary care networks*. London: The Health Foundation; 2019.
13. Smith JA, Parkinson S, Harshfield A, Sidhu M. *Early evidence of the development of primary care networks in England: a rapid evaluation study*. Southampton: NIHR Health Services and Delivery Research Topic Report; 2020. https://doi.org/https://doi.org/10.3310/hsdr-tr-129678
14. Checkland K, Hammond J, Warwick-Giles L, Bailey S. Exploring the multiple policy objectives for primary care networks: a qualitative interview study with national policy stakeholders. *BMJ Open* 2020;**10**:e038398. https://doi.org/10.1136/bmjopen-2020-038398
15. Morciano M, Checkland K, Hammond J, Lau Y-S, Sutton M. Variability in size and characteristics of primary care networks in England: observational study. *Br J Gen Pract* 2020;**70**:e899-e905. https://doi.org/10.3399/bjgp20X713441
16. . *Network Contract Directed Enhanced Services: Contract specification 2020/21 - PCN requirements and entitlements*; 2020.
17. Smith J, Mays N. Primary care organizations in New Zealand and England: tipping the balance of the health system in favour of primary care? *The International journal of health planning and management* 2007;**22**:3-19.

18. Lewis RQ. More Reform of the English National Health Service: From Competition Back to Planning? *International Journal of Health Services* 2018;**49**:5-16. https://doi.org/10.1177/0020731418797977

19. Scott C, Hofmeyer A. Networks and social capital: a relational approach to primary healthcare reform. *Health Research Policy and Systems* 2007;**5**:9. https://doi.org/10.1186/1478-4505-5-9

20. Mitchell SM, Shortell SM. The governance and management of effective community health partnerships: a typology for research, policy, and practice. *Milbank Q* 2000;**78**:241-89, 151. https://doi.org/10.1111/1468-0009.00170

21. Aunger JA, Millar R, Greenhalgh J, Mannion R, Rafferty A-M, McLeod H. Why do some inter-organisational collaborations in healthcare work when others do not? A realist review. *Systematic Reviews* 2021;**10**:82. https://doi.org/10.1186/s13643-021-01630-8

22. Aunger JA, Millar R, Greenhalgh J. When trust, confidence, and faith collide: refining a realist theory of how and why inter-organisational collaborations in healthcare work. *BMC Health Services Research* 2021;**21**:602. https://doi.org/10.1186/s12913-021-06630-x

23. Goodwin N, 6 P, Peck E, Freeman T, Posaner R. *Managing across diverse networks of care: lessons from other sectors*: Health Services Management Centre, University of Birmingham; 2004.

24. Kumpunen S, Natasha Curry, Tim Ballard, Hannah Price, Mike Holmes, Nigel Edwards. *Collaboration in general practice: surveys of GPs and CCGs*. Nuffield Trust and Royal College of General Practitioners; 2015.

25. Johl SK, Renganathan S. Strategies for gaining access in doing fieldwork: Reflection of two researchers. *Electronic Journal of Business Research Methods* 2010;**8**:42.

26. Tubaro P, Ryan L, D'angelo A. The visual sociogram in qualitative and mixed-methods research. *Sociological Research Online* 2016;**21**:1-18.

27. Gale NK, Heath G, Cameron E, Rashid S, Redwood S. Using the framework method for the analysis of qualitative data in multi-disciplinary health research. *BMC medical research methodology* 2013;**13**:117.

28. QSR International Pty Ltd. NVivo qualitative data analysis software. In.

29. NHS England and NHS Improvement. *Legislating for Integrated Care Systems: five recommendations to Government and Parliament*. London: NHS England and NHS Improvement; 2021.

30. Parkinson S, Smith J. Primary care networks in a time of pandemic. *BJGP Life* 2020.

31. . *COVID-19 vaccination programme 2020/21 – next steps*; 2020.

32. . *Update to GP contract arrangements for 2021/22*. London; 2021.

33. NHS SDO. *Networks Briefing: Key lessons for network management in healthcare*. London: National Co-ordinating Centre for NHS Service Delivery and Organisation Research and Development; 2004.

34. Pettigrew LM, Kumpunen S, Rosen R, Posaner R, Mays N. Lessons for 'large-scale' general practice provider organisations in England from other inter-organisational healthcare

For peer review only

collaborations. *Health Policy* 2019;**123**:51-61. https://doi.org/10.1016/j.healthpol.2018.10.017

35. Smith JA, Mays N. GP led commissioning: time for a cool appraisal. *BMJ* 2012;**344**:e980.
36. Fisher R, Smith J, Sidhu M, Parkinson S, Alderwick H. *NHS reform: Five key questions about the future of primary care networks in England*. London: The Health Foundation; 2021.
37. Carter R, Riverin B, Levesque J-F, Gariepy G, Quesnel-Vallée A. The impact of primary care reform on health system performance in Canada: a systematic review. *BMC Health Services Research* 2016;**16**:324. https://doi.org/10.1186/s12913-016-1571-7
38. Taylor CJ, Wright M, Jackson CL, Hobbs R. Grass is greener? General practice in England and Australia. *The British journal of general practice : the journal of the Royal College of General Practitioners* 2016;**66**:428-9. https://doi.org/10.3399/bjgp16X686377
39. Thorlby R, Smith J, Barnett P, Mays N. Primary care for the 21st century: learning from New Zealands independent practitioner associations. Research report. 2012.

For peer review only

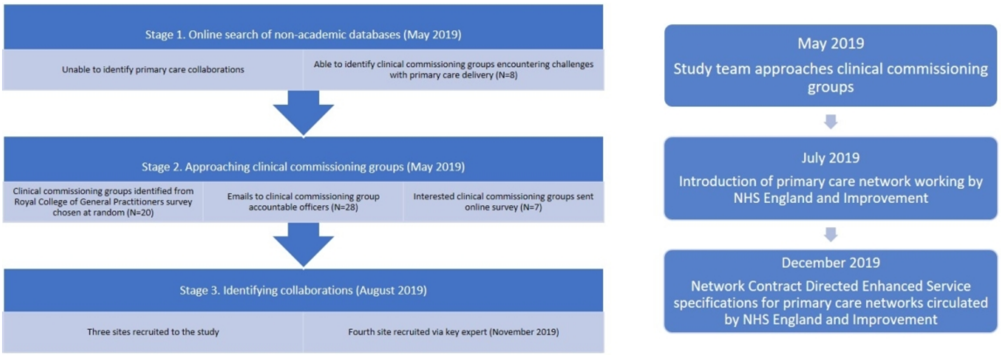

Figure 1: Summary of case study site sampling

253x89mm (150 x 150 DPI)

Revised Standards for Quality Improvement Reporting Excellence (SQUIRE 2.0)
September 15, 2015

Text Section and Item Name	Section or Item Description
**Notes to authors**	• The SQUIRE guidelines provide a framework for reporting new knowledge about how to improve healthcare<br><br>• The SQUIRE guidelines are intended for reports that describe system level work to improve the quality, safety, and value of healthcare, and used methods to establish that observed outcomes were due to the intervention(s).<br><br>• A range of approaches exists for improving healthcare. SQUIRE may be adapted for reporting any of these.<br><br>• Authors should consider every SQUIRE item, but it may be inappropriate or unnecessary to include every SQUIRE element in a particular manuscript.<br><br>• The SQUIRE Glossary contains definitions of many of the key words in SQUIRE.<br><br>• The Explanation and Elaboration document provides specific examples of well-written SQUIRE items, and an in-depth explanation of each item.<br><br>• Please cite SQUIRE when it is used to write a manuscript.
**Title and Abstract**	
**1. Title**	Indicate that the manuscript concerns an initiative to improve healthcare (broadly defined to include the quality, safety, effectiveness, patient-centeredness, timeliness, cost, efficiency, and equity of healthcare)
**2. Abstract**	a. Provide adequate information to aid in searching and indexing<br>b. Summarize all key information from various sections of the text using the abstract format of the intended publication or a structured summary such as: background, local problem, methods, interventions, results, conclusions
**Introduction**	*Why did you start?*
**3. Problem Description**	Nature and significance of the local problem
**4. Available knowledge**	Summary of what is currently known about the problem, including relevant previous studies

**5. Rationale**	Informal or formal frameworks, models, concepts, and/or theories used to explain the problem, any reasons or assumptions that were used to develop the intervention(s), and reasons why the intervention(s) was expected to work
**6. Specific aims**	Purpose of the project and of this report
**Methods**	*What did you do?*
**7. Context**	Contextual elements considered important at the outset of introducing the intervention(s)
**8. Intervention(s)**	a. Description of the intervention(s) in sufficient detail that others could reproduce it<br>b. Specifics of the team involved in the work
**9. Study of the Intervention(s)**	a. Approach chosen for assessing the impact of the intervention(s)<br>b. Approach used to establish whether the observed outcomes were due to the intervention(s)
**10. Measures**	a. Measures chosen for studying processes and outcomes of the intervention(s), including rationale for choosing them, their operational definitions, and their validity and reliability<br>b. Description of the approach to the ongoing assessment of contextual elements that contributed to the success, failure, efficiency, and cost<br>c. Methods employed for assessing completeness and accuracy of data
**11. Analysis**	a. Qualitative and quantitative methods used to draw inferences from the data<br>b. Methods for understanding variation within the data, including the effects of time as a variable
**12. Ethical Considerations**	Ethical aspects of implementing and studying the intervention(s) and how they were addressed, including, but not limited to, formal ethics review and potential conflict(s) of interest
**Results**	*What did you find?*
**13. Results**	a. Initial steps of the intervention(s) and their evolution over time (*e.g.*, time-line diagram, flow chart, or table), including modifications made to the intervention during the project<br>b. Details of the process measures and outcome<br>c. Contextual elements that interacted with the intervention(s)<br>d. Observed associations between outcomes, interventions, and relevant contextual elements<br>e. Unintended consequences such as unexpected benefits, problems, failures, or costs associated with the intervention(s).<br>f. Details about missing data
**Discussion**	*What does it mean?*
**14. Summary**	a. Key findings, including relevance to the rationale and specific aims<br>b. Particular strengths of the project

For peer review only

**15. Interpretation**	a. Nature of the association between the intervention(s) and the outcomes<br>b. Comparison of results with findings from other publications<br>c. Impact of the project on people and systems<br>d. Reasons for any differences between observed and anticipated outcomes, including the influence of context<br>e. Costs and strategic trade-offs, including opportunity costs
**16. Limitations**	a. Limits to the generalizability of the work<br>b. Factors that might have limited internal validity such as confounding, bias, or imprecision in the design, methods, measurement, or analysis<br>c. Efforts made to minimize and adjust for limitations
**17. Conclusions**	a. Usefulness of the work<br>b. Sustainability<br>c. Potential for spread to other contexts<br>d. Implications for practice and for further study in the field<br>e. Suggested next steps
**Other information**	
**18. Funding**	Sources of funding that supported this work. Role, if any, of the funding organization in the design, implementation, interpretation, and reporting

For peer review only

Table 2. Glossary of key terms used in SQUIRE 2.0. This Glossary provides the intended meaning of selected words and phrases as they are used in the SQUIRE 2.0 Guidelines. They may, and often do, have different meanings in other disciplines, situations, and settings.

Assumptions
Reasons for choosing the activities and tools used to bring about changes in healthcare services at the system level.

Context
Physical and sociocultural makeup of the local environment (for example, external environmental factors, organizational dynamics, collaboration, resources, leadership, and the like), and the interpretation of these factors ("sense-making") by the healthcare delivery professionals, patients, and caregivers that can affect the effectiveness and generalizability of intervention(s).

Ethical aspects
The value of system-level initiatives relative to their potential for harm, burden, and cost to the stakeholders. Potential harms particularly associated with efforts to improve the quality, safety, and value of healthcare services include opportunity costs, invasion of privacy, and staff distress resulting from disclosure of poor performance.

Generalizability
The likelihood that the intervention(s) in a particular report would produce similar results in other settings, situations, or environments (also referred to as external validity).

Healthcare improvement
Any systematic effort intended to raise the quality, safety, and value of healthcare services, usually done at the system level. We encourage the use of this phrase rather than "quality improvement," which often refers to more narrowly defined approaches.

Inferences
The meaning of findings or data, as interpreted by the stakeholders in healthcare services – improvers, healthcare delivery professionals, and/or patients and families

Initiative
A broad term that can refer to organization-wide programs, narrowly focused projects, or the details of specific interventions (for example, planning, execution, and assessment)

Internal validity
Demonstrable, credible evidence for efficacy (meaningful impact or change) resulting from introduction of a specific intervention into a particular healthcare system.

Intervention(s)
The specific activities and tools introduced into a healthcare system with the aim of changing its performance for the better. Complete description of an intervention includes its inputs, internal activities, and outputs (in the form of a logic model, for example), and the mechanism(s) by which these components are expected to produce changes in a system's performance.

Opportunity costs

For peer review only - http://bmjopen.bmj.com/site/about/guidelines.xhtml

Loss of the ability to perform other tasks or meet other responsibilities resulting from the diversion of resources needed to introduce, test, or sustain a particular improvement initiative

Problem
Meaningful disruption, failure, inadequacy, distress, confusion or other dysfunction in a healthcare service delivery system that adversely affects patients, staff, or the system as a whole, or that prevents care from reaching its full potential

Process
The routines and other activities through which healthcare services are delivered

Rationale
Explanation of why particular intervention(s) were chosen and why it was expected to work, be sustainable, and be replicable elsewhere.

Systems
The interrelated structures, people, processes, and activities that together create healthcare services for and with individual patients and populations. For example, systems exist from the personal self-care system of a patient, to the individual provider-patient dyad system, to the microsystem, to the macrosystem, and all the way to the market/social/insurance system. These levels are nested within each other.

Theory or theories
Any "reason-giving" account that asserts causal relationships between variables (causal theory) or that makes sense of an otherwise obscure process or situation (explanatory theory). Theories come in many forms, and serve different purposes in the phases of improvement work. It is important to be explicit and well-founded about any informal and formal theory (or theories) that are used.

For peer review only

The early development of primary care networks in the NHS in England: A qualitative mixed-methods evaluation

Journal:	*BMJ Open*
Manuscript ID	bmjopen-2021-055199.R1
Article Type:	Original research
Date Submitted by the Author:	17-Nov-2021
Complete List of Authors:	Parkinson, Sarah; RAND Europe, Health and Wellbeing<br>Smith, Judith; University of Birmingham, Health Services Management Centre<br>Sidhu, Manbinder; University of Birmingham, Health Services Management Centre
**Primary Subject Heading**:	General practice / Family practice
Secondary Subject Heading:	Health policy, Health services research, Qualitative research
Keywords:	HEALTH SERVICES ADMINISTRATION & MANAGEMENT, Health policy < HEALTH SERVICES ADMINISTRATION & MANAGEMENT, Organisation of health services < HEALTH SERVICES ADMINISTRATION & MANAGEMENT, PRIMARY CARE

I, the Submitting Author has the right to grant and does grant on behalf of all authors of the Work (as defined in the below author licence), an exclusive licence and/or a non-exclusive licence for contributions from authors who are: i) UK Crown employees; ii) where BMJ has agreed a CC-BY licence shall apply, and/or iii) in accordance with the terms applicable for US Federal Government officers or employees acting as part of their official duties; on a worldwide, perpetual, irrevocable, royalty-free basis to BMJ Publishing Group Ltd ("BMJ") its licensees and where the relevant Journal is co-owned by BMJ to the co-owners of the Journal, to publish the Work in this journal and any other BMJ products and to exploit all rights, as set out in our licence.

The Submitting Author accepts and understands that any supply made under these terms is made by BMJ to the Submitting Author unless you are acting as an employee on behalf of your employer or a postgraduate student of an affiliated institution which is paying any applicable article publishing charge ("APC") for Open Access articles. Where the Submitting Author wishes to make the Work available on an Open Access basis (and intends to pay the relevant APC), the terms of reuse of such Open Access shall be governed by a Creative Commons licence – details of these licences and which Creative Commons licence will apply to this Work are set out in our licence referred to above.

Other than as permitted in any relevant BMJ Author's Self Archiving Policies, I confirm this Work has not been accepted for publication elsewhere, is not being considered for publication elsewhere and does not duplicate material already published. I confirm all authors consent to publication of this Work and authorise the granting of this licence.

For peer review only

The early development of primary care networks in the NHS in England: A qualitative mixed-methods evaluation

Sarah Parkinson, RAND Europe, Westbrook Centre, Milton Road, Cambridge, CB4 1YG, UK

Judith Smith*, Health Services Management Centre, School of Social Policy, College of Social Sciences, University of Birmingham, 40 Edgbaston Park Rd, Birmingham, B15 2RT, UK

Manbinder Sidhu, Health Services Management Centre, School of Social Policy, College of Social Sciences, University of Birmingham, 40 Edgbaston Park Rd, Birmingham, B15 2RT, UK

***Corresponding Author,** Health Services Management Centre, School of Social Policy, College of Social Sciences, University of Birmingham, 40 Edgbaston Park Rd, Birmingham, B15 2RT, UK.

Email: j.a.smith.20@bham.ac.uk Tel: +44(0)121 414 7068

Word count excluding title page, abstract, references figures and tables: 5001

Abstract

Objectives: Primary care networks (PCNs) were introduced in the NHS in England in 2019 to improve integrated care for patients and help address financial and workforce sustainability issues in general practice. The purpose of this study was to collect early evidence on their implementation and development, including motivations to participate and what enables or inhibits progress. This paper considers the core characteristics of PCNs, and how this informs their management.

Design: A qualitative mixed-methods rapid evaluation was conducted across four case study sites in England, informed by a literature review and stakeholder workshop. Data collection comprised interviews, non-participant observation of meetings, an online survey and documentary review.

Results: General practitioners (GPs) are motivated to participate in PCNs for their potential to improve patient care, enable better coordinated services and enhance financial and workforce sustainability within primary care. However, PCNs also have an almost mandatory feel, based on the national policy context and significant financial incentives associated with joining them. PCNs offer potential to bring general practices together to work towards common goals, deliver national priorities and respond rapidly to local needs.

Conclusions: PCNs face similar challenges to other meso-level primary care organisations internationally, as they respond to local and national priorities and operate in a context of multiple goals and interests. In managing these organisations, it is important to find a balance between local and national autonomy, decision making and control.

For peer review only

Strengths and limitations of this study

- As a rapid evaluation, this study responds to current policy-relevant questions about the early development of PCNs, and developments in how primary care is delivered in the NHS in England.
- The qualitative approach provides insights into why GPs participate in PCNs and the experience of implementation.
- The mixed-methods approach to this evaluation allows data to be triangulated between sources and ensures that a broad range of perspectives is captured.
- The use of a theoretical framework to interpret the findings from this evaluation helps contextualise them within the wider literature, and understand what this evaluation means for other meso-level primary care organisations internationally.
- This evaluation provides an insight into the early development and implementation of PCNs, along with information about their initial response to the COVID-19 pandemic. Although the study reflects on how PCNs will continue to develop, for example in response to new policies in the English NHS, definitive conclusions about the impact of PCNs were outside the scope of this study.

Conflict of interest

No conflicts of interest are declared.

Ethical approval

An application for ethical review to the University of Birmingham's Research Ethics Committee was made by the project team and approval was gained in July 2019 (ERN_13-1085AP34).

Funding sources

This project was funded by part of a grant from The National Institute for Health Research, Health Services and Delivery Research programme (HSDR 16/138/31 – Birmingham, RAND and Cambridge Evaluation Centre).

Data availability statement

Due to the consent process for data collection at case study sites within this evaluation, there are no data that can be shared.

Acknowledgements

We thank Amelia Harshfield, who contributed to the conception and design of the study, and data collection at two study sites. We also thank Natasha Elmore, Dr Sarah Ball, and Jon Sussex (all from RAND Europe) for their contribution to data collection, gaining ethical approval, and providing project advice; Dr Rebecca Fisher (Senior Policy Fellow, Health Foundation) and Dr Mina Gupta (GP, Modality Partnership) for peer review of our online surveys; Samantha Hinks (NHS England and

For peer review only

Improvement) and Professor Katherine Checkland (University of Manchester) for their on-going advice and sharing learning from their own evaluations and research throughout; Professor Russell Mannion (University of Birmingham), Dr Katie Coleman and Mark Platt (BRACE Centre Health and Care Panel) who reviewed our study protocol; and Professor Justin Waring (University of Birmingham) and Dr Anna Dixon (formerly Chief Executive, Centre for Ageing Better) for undertaking critical review of our findings.

Author contributions

JS was principal investigator for the study and responsible for its conception, design, conduct and writing up, with support from MS and SP. MS, SP, and JS undertook data collection and SP led data analysis supported by JS and MS. SP wrote the first draft of this paper, and MS and JS commented on and contributed to drafts.

For peer review only

INTRODUCTION

Primary care networks (PCNs) are the latest in a long line of general practice (GP) collaborations in the NHS in England dating back to GP fundholding and locality commissioning in the 1990s.[1] Predecessor collaborations have encompassed a wide range of arrangements, including total purchasing projects, primary care groups and trusts, practice-based commissioning, personal medical services schemes, and clinical commissioning groups.[2-4] These collaborations have ranged from informal networks to formal multi-site practice organisations and super-partnerships where GP practices merge important functions such as managing finances and contracts.[5] These other forms of collaborations have had varied aims, including improving care at a local level and delivering new services to patients, strengthening the resilience of general practice, and supporting better management in primary care, including improved financial stability.[2, 6]

PCNs were introduced in 2019 as part of the NHS Long Term Plan,[7] which claimed that these new networks would create integrated and community-based healthcare, support expanded neighbourhood teams, increase workforce sustainability and deliver on a number of national priorities such as health inequalities and early cancer diagnosis. The NHS Long Term Plan announced that at least £4.5 billion would be invested in these networks over the following five years. Since this time, nearly all practices in England have joined a PCN.[8] PCNs were introduced into the English NHS at a time of particular financial and workforce sustainability challenges in primary care and general practice,[9-11] which is important in understanding their goals and policy context. Key characteristics of PCNs are set out in Box 1 below.

One notable way in which PCNs depart from some previous forms of collaborative working is that many prior collaborations (for example, GP super-partnerships, GP federations, GP multifunds) evolved from the ground-up, meaning that local actors within primary care had taken the initiative to work together out of necessity or shared interest. In contrast, PCNs have been encouraged through national policy with significant financial incentives,[12] giving them a compulsory, top-down feel when compared to some previous forms of collaborative working, although they share this more mandated approach with primary care groups, primary care trusts, and practice-based commissioning.[13] While participation in PCNs is in theory voluntary for GP practices, in reality almost all practices have interpreted them as mandatory, considering the significant levels of new funding that are distributed through PCNs.[14]

For peer review only

Box 1: Key features of PCNs

- PCNs are intended to bring together groups of neighbouring (geographically contiguous) GP practices, along with other primary care providers such as community pharmacies, dentists, optometrists and voluntary sector organisations.
- The NHS Long Term Plan specified that PCNs should cover a patient population of 30,000 to 50,000 patients,[7] although many now cover much larger populations (upwards of 100,000 to 150,000 patients)[15]
- PCNs receive funding on a per-patient basis for enhanced services and additional funding to support recruiting new shared roles such as social prescribers and clinical pharmacists. This funding is distributed through their local clinical commissioning group (CCG, the local funding agency which commissions most hospital, mental health and community services in local areas in England).
- Each PCN is led by a clinical director, who receives funding for their role depending on population size of the network, weighted by deprivation and burden of morbidity.[14]
- The Direct Enhanced Services (DES) contract[16] specifies what services PCNs must provide to gain access to funding, and includes specifications for structured medication reviews, general practice support of care homes, anticipatory care for patients in the community with complex needs, early cancer diagnosis services, cardiovascular health, and health inequalities. The contract sets out what local commissioners of primary care services must offer to providers participating in PCNs, and what services PCNs must deliver to receive additional funding.

Primary care networks are meso-level organisations,[6] operating between formal funders or commissioners, and local GP practices. As such, they are somewhat hybrid in nature, being both national and local, and extrinsically (e.g. based on policy and incentives) and intrinsically motivated (e.g. based on expected benefits and desire to collaborate) through a national policy initiative as well as shared goals and interests. As meso-level organisations, PCNs share characteristics with international experiences of primary care organisations, displaying complexity in their form, objectives and ways of working,[14] and occupying a sometimes unclear position within national and local healthcare systems.[17]

As networks of healthcare professionals, there is also much to learn about PCNs from prior work on the characteristics of professionally-led networks and healthcare network management. The existing literature explores effective ways to manage and govern networks in healthcare, depending on the structure of the network and the context within which the network is functioning.[18-22] This paper contributes to this body of literature, and applies existing theoretical work on healthcare networks management to the early experience of PCNs in the NHS in England.

This analysis draws on a rapid mixed methods evaluation of the first year of operation of PCNs[13] to explore their implementation and early progress. The findings are interpreted using theory about the nature of health care network structure and management, drawn from work by Goodwin et al. (2004).[23] In particular, this analysis includes an examination of the characteristics that PCNs share with ‘enclave networks’, with a rather flat organisational structure, formed of relatively close-knit groups of professionals and seeking to have a bottom-up and locally-owned sense of purpose, as well as ‘hierarchical networks,’ designed to undertake specific tasks as dictated through contractual and funding mechanisms that are enacted in a top-down manner on behalf of a national health system.[23]

This analysis addresses the following questions:

For peer review only

- RQ1: What was the rationale for GP practices to join and participate in a PCN?
- RQ2: What enabled or inhibited the early progress made by PCNs?
- RQ3: What are the core characteristics of PCNs, given their role as meso-level organisations working between local general practice and national health funders and commissioners?
- RQ4: What does this experience reveal about how to manage and prepare meso-level primary care collaborations to fulfil local and national policy expectations?

METHODS

The rapid evaluation study explores four case study sites across England through documentary review, interviews, a survey, and non-participant observations, which are described below. The full findings from this research are described in Smith, et al. (2020),[13] while this article draws on additional analysis of evaluation data to address the research questions set out above, and as detailed in Table 1.

Table 1: Summary of methods and research questions

Study phase	Description	Research questions
Rapid evidence assessment	An overview of published evidence to distil prior learning and inform the development of propositions to be tested through comparative case studies of new primary care networks	RQ1 and RQ2
Stakeholder workshop	A workshop led by members of the study team for relevant stakeholders (e.g. academic and policy experts in the field, patient and public involvement representatives). The aim of this workshop was to clarify evidence gaps and evaluation questions of particular relevance to emerging policy on primary care networks and thus inform next steps.	RQ1 and RQ2
Comparative case studies of four primary care networks	Interviews with those involved in the conceptual design, implementation of primary care networks in their respective sites, and exploration of relationship with any prior GP collaboration in the case study site; analysis of key documentation (both internal and publicly shared); non-participant observation of strategic meetings; and an	RQ1, RQ2, RQ3 and RQ4

For peer review only

	online survey to collate information on challenges associated with collaborative working and measuring early impacts.	
Analysis of findings from case studies to develop a nuanced understanding of the development of primary care networks in the NHS in England	Share and discuss findings generated from data collection from case studies.	RQ1, RQ2, RQ3 and RQ4

Patient and Public Involvement

A half-day project design workshop was undertaken in November 2018 and involved, in addition to the research team, national primary care policy officials, a patient representative, academics with experience of researching primary care organisations, and policy experts in the field (N=12). The aim of the workshop was to help identify gaps in the literature, and thereby devise relevant research questions. Participants at the workshop felt a key unexplored area was the experiences of primary care collaborations in rural, as opposed to urban areas, to better understand regionally-specific challenges in primary care. Furthermore, attendees were keen for researchers to investigate sites where it had proved challenging to sustain primary care collaborations, and to examine what management and organisational development skills and capacity are needed to make a PCN work effectively.

Sampling and recruitment of case study sites

Purposive sampling was used to select sites that had not been involved in research studies or evaluations over the previous two years, and to ensure that the sample included rural sites, as well as collaborations that had previously faced challenges in sustaining collaborative working. Potential clinical commissioning groups (CCGs) to approach were identified using a combination of an online search of grey literature and those that had responded to a 2017 study by the Royal College for General Practitioners.[24] Twenty-eight CCGs were contacted as potential participants from May to August 2019, and those that responded (n=7) were sent a short survey to determine whether emerging PCNs in their area met the inclusion criteria. Three case study sites were identified using this approach, and a fourth was identified through engagement with providers known to the researchers. Three case study sites were PCNs, and one was a super-partnership with member practices also belonging to several PCNs. A short description of the four case study sites is provided in Table 2 below and a summary of the sampling approach is illustrated in Figure 1.

For peer review only

Table 2: Description of case study sites

Case study site	Short description
Site A	PCN in a rural setting covering a patient population of 75,000 (large ageing population, mostly White British), where practices had previously worked together through an informal model of locality working. Some practices in the PCN were also involved in a super-partnership.
Site B	Super-partnership in a rural setting covering a patient population of 130,000 patients (large ageing population, mostly White British). Practices within super-partnership were part of four separate PCNs which also contained non-super-partnership practices.
Site C	PCN in an urban and semi-urban setting, covering a patient population of about 60-70,000 patients (socioeconomically disadvantaged population, significant Black, Asian and Minority Ethnic population), where practices had previously worked together formally in a GP Neighbourhood.
Site D	PCN in a rural setting, covering a population of 30,000 patients (large ageing population, mostly White British), where practices previously worked together and with community teams informally.

Data collection and recruitment

Data collection was facilitated through a gatekeeper,[25] or contact point, at each case study site. A total of 29 semi-structured interviews with 25 participants were conducted using a topic guide (summarised in Box 2), each lasting between 30-60 minutes. Participant characteristics are described in Table 3. A minimum of one and a maximum of nine interviews were conducted at each of the four case study sites with both clinical and non-clinical staff, mainly with those in leadership or management positions within the PCN. Interviews were audio recorded, transcribed verbatim using a professional transcription service and pseudonymised. Four of these interviews were follow-up interviews with PCN managers to gather information on their response to Covid-19. Interviews were completed both face-to-face and virtually (due to the onset of the Covid-19 pandemic) by JS, SP, MS and two further researchers with qualitative interviewing experience. Data saturation was achieved for themes regarding rationale for GP practices to join and participate in a PCN and what may have inhibited or enabled progress; although saturation may not have been achieved for themes focused on the trajectory of primary care networks in the post-pandemic English NHS given that data collection ended in the initial phase of the pandemic.

Nine meetings (e.g. board- or partner-level meetings, task group meetings) were observed across the four case study sites by SP, MS and two other researchers with experience in non-participant observations. A template was used to take notes at each meeting on the topics discussed and dynamics within each case study site, including a sociogram to visualise how meeting participants interacted with one another.[26] For both interviews and observation, participants were given an information sheet about the study, given the opportunity to ask questions, and provided informed consent prior to data collection. Lastly, gatekeepers provided access to key documents at each site, including material related to the structure of the PCN and any pre-existing GP collaboration, governance and decision-making, agendas of previous meetings and local communication activities. Information was extracted from these documents using a structured Excel template based on the aims of the evaluation.

For peer review only

Box 2: Interview topics

1. Models of GP collaboration in the local area and how previous and extant collaborations relate to PCNs
2. Specific challenges to PCN working, particularly in relation to urban and rural settings, and any practice that may have left PCNs
3. How collaborative working in local primary care systems has evolved since introducing PCNs
4. Nature of professional relationships within PCN
5. Motivations to participate in PCNs
6. Key goals and outcomes for short and medium to long term for PCNs
7. Early impacts of PCNs

Table 3: Characteristics of interviewees from four case study sites

**Site**	**Description**	**Number (N)**
Site A	Primary care clinical staff	4<br>(Int1-4)
	Primary care non-clinical staff	5<br>(Int5-9)
Site B	Primary care clinical staff	3<br>(Int10-12)
	Primary care non-clinical staff	3<br>(Int13-15)
	Clinical commissioning group staff	2<br>(Int16-17)
Site C	Primary care clinical staff	4<br>(Int18, 20-22)
	Primary care non-clinical staff	2<br>(Int23-24)
	Clinical commissioning group staff	1<br>(Int19)
Site D	Primary care non-clinical staff	1<br>(Int25)
**Total**		**25**

Synthesis and analysis

After data had been collected, the evaluation team (JS, SP and MS) participated in a half-day data analysis workshop to review data collected, discuss themes and begin systematic analysis of the data

For peer review only

as per the framework method for data analysis described in Gale et al. (2013).[27] Data from interviews were analysed through deductive coding with NVivo 12 software[28] using a codebook that had been developed by the evaluation team based on the evaluation aims, available literature on primary care collaborations and initial reading of interview transcripts. Analysis was led by SP, whereby an initial coding frame was developed based on codes arising from a sample of five transcripts by MS and SP. MS and SP coded all transcripts, and further developed codes based on subsequent transcripts and further discussions. This approach was also applied to data from non-participant observation meeting notes and documentary review template. After analysis, themes were discussed in a second half-day workshop (JS, SP, and MS) with the evaluation team to synthesise evidence for each of the research questions and develop an overarching narrative summary (written by JS) of the findings.

FINDINGS

Analysis of data from evaluation fieldwork highlights the rationale for general practices to join PCNs, what has facilitated and inhibited the early progress of these new networks, and what this means about the nature of how PCNs operate and are likely to develop longer term. The full findings of the evaluation are reported elsewhere,[13] while this secondary analysis of data from the evaluation focuses on interpreting the data in relation to health care network structure and management.

Reasons for joining and participating in a PCN

There are many reasons why GP practices join and participate in PCNs, these being based on both top-down and bottom-up motivations. These reasons reflect the policy and incentive structure that led to the introduction of local PCNs within the national context of the English NHS, as well as a genuine desire to collaborate locally to ensure the sustainability of primary care and improve and enhance the services available to patients.

When asked about the reasons why their practice joined a PCN, interviewees involved in practice-level management reflected that PCNs are, in effect, perceived to be mandatory given the sizeable financial incentives associated with PCN membership. There was some sense of frustration about the perception that practices have been forced or coerced into joining PCNs, although others asserted that the national PCNs policy is based on the known efficacy of primary care in responding to incentives.

> *'Most my GP colleagues in other practices and within my partnership, we all were very suspicious of it and also didn't feel it was the right mechanism for delivering the resilience in general practice which we need because it was being foisted on us… it was the only way we could see that we were going to get any new money coming into general practice…. I guess we thought… we might as well.'* (Int1)

Despite this focus on top-down motivations, bottom-up motivations also contributed to the desire to join a PCN. All four case study sites had a history of their general practices collaborating with one another to some extent, either through informal groupings or more formal arrangements such as super-partnerships or locality forums. Respondents involved in the management of PCNs reflected that practices typically collaborate to fill gaps in the services that single practices are able to provide, and to facilitate GPs working with community health teams, social services and the voluntary sector to provide extended care that addresses local population health needs.

> *'We're only a small network, 35,000 patients in the network… I sort of see the 35,000 rather than the 3,000 we've got on our list. So I'm really enthusiastic, and I want to make sure that the 35,000 are looked after, as much as my 3,000'*

For peer review only - http://bmjopen.bmj.com/site/about/guidelines.xhtml

A clear desire to improve the sustainability of primary care also was a shared goal that motivate GP practices to work with one another within PCNs. Some interviewees also mentioned that working in collaboration across practices is attractive because of the potential for financial efficiency and sustainability by sharing back office functions, reducing duplication of administrative tasks, introducing more robust financial management processes and making it easier to recruit and retain new staff, for example, by providing more opportunities for training, education and specialisation.

The reasons for joining and participating in PCNs impact not only on individual GP practices, but also the structure of PCNs themselves. Networks can be built on the shared interests, goals and motivations of members, and also through formalised structures and top-down regulation that require or incentivise membership. In the case of PCNs, members are bonded by a blend of these structural mechanisms. National policy has prompted the forming of PCNs, but in the absence of national policy incentives, it would remain in GP practices best interests to still collaborate with one another to provide services, improve management and realise efficiencies based on their mutual interests. This blend of motivations influences the relationship that network members have with one another, and also the place of the network within the wider health and care system.

Local engagement and ownership of PCNs

Engagement by practices in the PCN at a local level is critical to ensuring that networks not only deliver the national priorities set for them, but also address local health needs and improve the integration of services across primary care. This is of particular importance given NHS policy direction towards new integrated care systems.[29]

Early in the implementation of PCNs, there tended to be little engagement with the PCN below the leadership and management level among staff in constituent GP practices. At this stage, there seemed to be a sense that PCNs had not yet had much effect, and that local practices would continue to deliver services for patients and operate much as they did before PCNs.

> *'Some of the staff wouldn't know that we were in a network, even though we've told them about it. If you then said about the PCN, they'd say well what's that?'* (Int6)

In some cases, this lack of engagement was reported to be exacerbated by a perception that PCNs were the latest in a long line of collaborative mechanisms set out by the NHS for GPs. Frustration was expressed about frequently changing NHS policy that disrupts extant ways of working, including activity under way to improve patient care through other forms of locally developed primary care collaborations such as federations and super-partnerships. There was also some irritation expressed by interviewees and observed in meetings around the prescriptive nature of the services required by the DES contract,[16] which further tempers local buy-in to PCNs, particularly where services specified in the DES contract are perceived as not tailored to the needs and preferences of local populations.

> *'We just thought, well we've been there before. We deal with the box ticking. Get the box ticking done and then deliver what… might improve care for our patients'* (Int1)

There was also genuine enthusiasm expressed by some interviewees for PCNs as a sign of greater investment in NHS primary care, and as a way to raise the collective voice of GPs and primary care, for example in terms of negotiating collective contracts. Some of those involved in the leadership and management of PCNs expressed that they have experienced a sense of empowerment in working on something larger than a single practice, and being involved in strategic planning of local primary and community health services over and above single-practice working.

For peer review only

'The main thing that has come in – and this isn't just here – is the enthusiasm with which mostly a new set of GP faces have really taken on a new role and are invigorated and believe they're a bit empowered, and they're doing something at a bigger, more strategic level than out of practice' (Int19)

Where PCNs are perceived as a continuation of existing efforts to improve general practice sustainability and local healthcare, there seems to be a high level of enthusiasm and buy-in. However, where PCNs are perceived as a disruption to previous ways of working and a divergence from the goals of pre-existing forms of GP collaboration, there seem to be tensions and frustrations. On balance, engagement and buy-in will need to be fostered in order to build support for PCNs among wider primary care teams, and to ensure that those involved in managing and leading PCNs remain dedicated to their success.

The level of local engagement with and ownership of PCNs is connected to how they are structured as networks. Where PCNs are felt locally to be part of existing efforts to improve care, population health management and practice management, more individuals within GP practices appear to have bought into the premise of network working. In turn, the network is perceived to be founded upon shared goals and interests, and less on top-down mechanisms that contractually bind network members together. However, the opposite is also true. Where PCNs are thought to be another top-down policy change, fewer individuals buy into the idea of primary care networks, and there is likely to be increasing frustration about top-down interruptions to existing ways of working at a local level.

The role of PCNs in the local health system

This evaluation explored the first year of the development and implementation of PCNs, as they were still finding their place within the wider health and social care system. Different local contexts, for example relationships with statutory NHS bodies and histories of previous collaborative working, contributed to a diversity of ways in which PCNs have been working within local healthcare systems. As the COVID-19 pandemic emerged in 2020, this also influenced the role of PCNs within the local and wider NHS.

One way in which this variation played out was through the relationship between PCNs and local clinical commissioning groups (CCGs, which commission most hospital and community services in local areas in England). Some PCNs had drawn on management support from the local CCG throughout their development and implementation, while other PCNs reported little involvement from the CCG, or even cases of tension where the CCG was perceived as exerting undue influence over PCN priorities and budgets.

Variation in local context was also evident in the relationship between PCNs and pre-existing forms of GP collaboration, including GP federations and super-partnerships. At times, PCNs had been able to build on good working relationships established from previous collaborative working between practices and with other parts of the health and social care system and voluntary sector, which helped establish the position of PCNs locally. In one case study site, the super-partnership exerted considerable influence on PCNs to which member practices belonged, to the extent that PCNs merged and expanded to fit the geographical boundary of the super-partnership. These shifts will inevitably affect an individual PCN's place within the local health and care system and the scale at which the PCN operates in terms of its patient population.

Lastly, the COVID-19 pandemic has further shaped the place of PCNs within local and national health and social care systems.[30] PCNs have been an important mechanism in delivering the national

For peer review only

COVID-19 vaccination programme and have led the designation and deployment of vaccination sites after being asked to do so by NHS England and Improvement in December 2020.[31] Locally, PCNs were key to organising the delivery of primary care during the pandemic, for example by organising 'hot' and 'cold' hubs to care for COVID-19 and non-COVID-19 patients, and helping to coordinate the movement of staff between practices.[13] PCNs' role in both national and local healthcare delivery during the pandemic has already influenced their role within the health and social care system (e.g. by influencing national priorities that PCNs will focus on, including long COVID and weight management[32]), in ways that will likely become clearer as England emerges from the pandemic.

The place of PCNs within the wider health and care system is also linked to how they are structured and gain legitimacy as networks. Depending on the PCN's relationship with other organisations locally and nationally (e.g. with CCGs and local super-partnerships), and the demands being placed on PCNs due to system-level pressures (e.g. the pandemic), the place of the PCN within the wider system shifts. At times, the PCN is a mechanism for collaboration on certain, specified tasks, while at other times, it is a primary unit to deliver critical tasks such as primary care's pandemic response, and a focal point for interaction between local primary care and wider systems.

DISCUSSION

This evaluation reveals that PCNs, while introduced through national policy, are also based on shared goals of improving sustainability in primary care and improving integrated services for patients. While they are organised around delivering a set of priorities set out in the national DES contract,[16] they are also firmly based in local health and care systems, dependent on their local context and population health needs. Beyond their initial development and implementation, a challenge for PCN will be to build buy-in and engagement and clarify their place within the wider health and care system. To support PCNs as they continue to develop, and to ensure they are able to address both national priorities and local health population needs, including health inequalities, it will be important to ensure that appropriate management structures are in place, while also giving PCNs sufficient autonomy to adapt.

Although PCNs specifically are unique to the English NHS, thinking about what support they are likely to need to address local and national priorities longer term is informative for wider discussion of the international experience of meso-level primary care organisations. Primary care organisations in other jurisdictions find themselves, like PCNs, shifting between a focus on local and national health priorities, and face challenges finding their place in wider health and care systems. They also report the common risk of being swept into increasingly centralised functions such as those identified in national policy initiatives.[17]

Goodwin et al. (2004)[23] provide a lens for thinking about the kind of management and support that PCNs and similar international examples of primary care organisation may need to ensure that they can reach their full potential. The authors establish a typology of three types of networks, based on the level of social regulation and social integration within the network (see Table 4).

Table 4: Different networks structures – Adapted from NHS SDO (2004),[33] based on Goodwin et al. (2004)[23]

Network type	Key characteristics	Key lessons for network management

For peer review only

**Enclave**	• High social regulation and low social integration<br>• Equality between members, flat internal structure<br>• High level of social cohesion and share commitment to common interests, values and goals	• Creates bottom-up legitimacy and promotes creation of new ways of working<br>• May fail when motivation of members is exhausted or when tensions occur<br>• Management may be administrative, helping to facilitate collaborative working, but without formal audits
**Hierarchical**	• High social regulation and high social integration<br>• Centred around organisational core that is able to regulate its members<br>• May be sustained by common interests, values and goals, but also based on structured agreements and protocols	• Most successful in coordinating and executing pre-defined tasks<br>• May fail through over-regulation, which limits ability to innovate and leads to low motivation of members<br>• Management to coordinate defined activities and provide central direction, although it is suggested that mandated networks should be avoided
**Individualistic**	• Low social regulation and low social integration<br>• Single entities or organisations that come together to achieve certain tasks<br>• No strong sense of shared interests, values and goals	• Innovative and flexible, with fluid membership<br>• May fail due to high cost of membership, competition and conflict between members that can limit desire to work jointly<br>• Management may help set targets, incentives and monitoring activities

PCNs can be understood both as enclave and hierarchical networks. They are simultaneously founded on shared goals and motivations and a relatively flat structure whereby each practice within the PCN has a voice, as well as being organised to be able to execute pre-defined tasks specified in the DES contract based on the national policy and funding infrastructure that initiated and surrounds them. Examining PCNs through this theoretical lens allows a more nuanced approach to the support that PCNs will require going forward, including in addressing the issues that PCNs face in terms of securing local ownership and engagement, and clarifying their role within the wider health and social care system.

As enclave networks, PCNs share the common goal of wanting to ensure sustainability in primary care, including financial and workforce sustainability, and improving integrated services that meet the needs of the patients of constituent practices. Locally, there is a preference for focusing on the characteristics that PCNs share with enclave networks, as evidenced by the enthusiasm and commitment that was expressed for the underlying goals of PCNs and the ability to work collaboratively to address local population health needs, as compared to the reticence and frustration towards the top-down, prescriptive nature of PCN policy, particularly where they were not perceived to be aligned with local priorities. Fostering this sense of shared goals and intrinsic motivation may help encourage buy-in and engagement with PCNs, and allow them the space and autonomy to arrive

For peer review only

at solutions that address local population health needs. Even as PCNs continue to address national health priorities and complete pre-defined tasks, it will be important to balance and align these with local priorities to foster buy-in, engagement and a shared sense of interests and goals within PCNs.

PCNs also share characteristics with hierarchical networks – they emerged from a centrally-determined policy and funding mechanism, and are designed to deliver services as set out in the national specification for PCNs.[16] In this sense, PCNs are well-suited to deliver on pre-determined tasks and respond to direction and guidance from central bodies, and have been effective in quickly making progress towards national strategic goals by establishing new and enhanced services for patients. However, as hierarchical networks they face a risk of over-regulation and excessive performance management that could inhibit motivation and enthusiasm for PCN teams and hamper their ability to innovate locally, which has been an issue for predecessor primary care organisations in the past.[34, 35]

The risk of over-regulation will be especially important to consider as the proposed integrated care systems (ICSs) are implemented nationally, CCGs are abolished, and PCNs likely find themselves having to work out their role within a restructured NHS.[36] PCNs have been identified as critical to the future success of ICSs by NHS England and Improvement and the Department of Health and Social Care,[29] which will likely have implications in terms of how PCNs are organised. It is possible that PCNs will come under pressure to grow in size and complexity, merge with neighbouring PCNs, which will add to the challenges they face in terms of local engagement if these risks are not carefully mitigated. The risk that PCNs are increasingly drawn into formal hierarchical arrangements and mergers is a common experience among meso-level organisations in primary care in the international context.[37-39]

CONCLUSION

This evaluation reveals that PCNs demonstrate significant potential to swiftly deliver new services to patients, respond to national priorities, bring together primary care providers with common motivations and interests, and improve financial and workforce sustainability in primary care. Furthermore, during the pandemic PCNs have responded to both national priorities in their participation in England's vaccination programme, as well as rapidly responding to local needs, for example by coordinating the movement of staff and patients between 'hot' and 'cold' hubs.

The task ahead for PCNs will be to ensure that they are able to address national priorities that are centrally defined, as well as adapting to fit local health needs. Focusing on the shared goals that motivate GP practices to want to collaborate with one another, and protecting PCNs from over-regulation, will be especially important as PCNs find their place within the wider NHS as it emerges from the pandemic, and as integrated care systems (ICSs) are implemented.

Primary care organisations like PCNs are often strongly placed to address local and national needs, being both enclave and hierarchical in nature, and should continue to address both of these areas. Careful attention needs to be paid to how these priorities are balanced, and how decisions are made that shape how these organisations fit into wider health and care systems. In order to enable these organisations to reach their full potential, the core characteristics of these organisations must be considered in deciding how they should be managed, including the motivations why individual providers join these organisations and the policy context that led to their development.

For peer review only

REFERENCES

1. Smith J, Harshfield A. Primary care networks: a marathon not a sprint. *Health Services Journal* 2019. https://www.hsj.co.uk/primary-care/primary-care-networks-a-marathon-not-a-sprint/7025889.article.
2. Pettigrew L, Nicholas Mays, Stephanie Kumpunen, Rebecca Rosen. *Large-scale general practice in England: what can we learn from the literature?* Nuffield Trust; 2016. https://www.nuffieldtrust.org.uk/research/large-scale-general-practice-in-england-what-can-we-learn-from-the-literature.
3. Exworthy M, Mannion R, Powell M. *Dismantling the NHS?: Evaluating the impact of health reforms.* Policy Press; 2016. doi:10.1332/policypress/9781447330226.001.0001.
4. Alderwick H, Dunn P, McKenna H, Walsh N, Ham C. *Sustainability and transformation plans in the NHS: how are they being developed in practice?* London: The King's Fund; 2016. https://www.kingsfund.org.uk/publications/stps-in-the-nhs.
5. Rosen R, Stephanie Kumpunen, Natasha Curry, Alisha Davies, Luisa Pettigrew, Lucia Kossarva. *Is bigger better? Lessons for large-scale general practice*. Nuffield Trust: ISBN: 978-1-910953-12-9; 2016. https://www.nuffieldtrust.org.uk/research/is-bigger-better-lessons-for-large-scale-general-practice.
6. Smith J, Goodwin N. *Towards managed primary care: the role and experience of primary care organizations*: Ashgate Publishing, Ltd.; 2006.
7. NHS England. *The NHS Long Term Plan*; 2019. https://www.longtermplan.nhs.uk/publication/nhs-long-term-plan/.
8. Baird B. *Primary care networks explained*. London: The King's Fund; 2019. https://www.kingsfund.org.uk/publications/primary-care-networks-explained.
9. Charlesworth A, Gershlick B, Firth Z, Kraindler J, Watt T. *Investing in The NHS long term plan: Job done?* London: The Health Foundation; 2019. https://www.health.org.uk/publications/reports/investing-in-the-nhs-long-term-plan.
10. Fisher R, Turton C, Gershlick B, Alderwick H, Thorlby R. *Feeling the strain: What The Commonwealth Fund's 2019 international survey of general practitioners means for the UK*. London: The Health Foundation; 2020. https://www.health.org.uk/publications/reports/feeling-the-strain.
11. Nelson P, Martindale A-M, McBride A, Checkland K, Hodgson D. Skill-mix change and the general practice workforce challenge. *Br J Gen Pract* 2018;**68**:66. https://doi.org/10.3399/bjgp18X694469
12. Fisher R, Thorlby R, Alderwick H. *Briefing: Understanding primary care networks*. London: The Health Foundation; 2019. https://www.health.org.uk/publications/reports/understanding-primary-care-networks.
13. Smith JA, Parkinson S, Harshfield A, Sidhu M. *Early evidence of the development of primary care networks in England: a rapid evaluation study*. Southampton: NIHR Health Services and Delivery Research Topic Report; 2020. https://doi.org/https://doi.org/10.3310/hsdr-tr-129678
14. Checkland K, Hammond J, Warwick-Giles L, Bailey S. Exploring the multiple policy objectives for primary care networks: a qualitative interview study with national policy stakeholders. *BMJ Open* 2020;**10**:e038398. https://doi.org/10.1136/bmjopen-2020-038398

15. Morciano M, Checkland K, Hammond J, Lau Y-S, Sutton M. Variability in size and characteristics of primary care networks in England: observational study. *Br J Gen Pract* 2020;**70**:e899-e905. https://doi.org/10.3399/bjgp20X713441

16. NHS England and NHS Improvement. *Network Contract Directed Enhanced Services: Contract specification 2020/21 - PCN requirements and entitlements*; 2020. https://www.england.nhs.uk/wp-content/uploads/2020/03/Network-Contract-DES-Specification-PCN-Requirements-and-Entitlements-2020-21-October-FINAL.pdf.

17. Smith J, Mays N. Primary care organizations in New Zealand and England: tipping the balance of the health system in favour of primary care? *The International journal of health planning and management* 2007;**22**:3-19. doi: 10.1002/hpm.866

18. Lewis RQ. More Reform of the English National Health Service: From Competition Back to Planning? *International Journal of Health Services* 2018;**49**:5-16. https://doi.org/10.1177/0020731418797977

19. Scott C, Hofmeyer A. Networks and social capital: a relational approach to primary healthcare reform. *Health Research Policy and Systems* 2007;**5**:9. https://doi.org/10.1186/1478-4505-5-9

20. Mitchell SM, Shortell SM. The governance and management of effective community health partnerships: a typology for research, policy, and practice. *Milbank Q* 2000;**78**:241-89, 151. https://doi.org/10.1111/1468-0009.00170

21. Aunger JA, Millar R, Greenhalgh J, Mannion R, Rafferty A-M, McLeod H. Why do some inter-organisational collaborations in healthcare work when others do not? A realist review. *Systematic Reviews* 2021;**10**:82. https://doi.org/10.1186/s13643-021-01630-8

22. Aunger JA, Millar R, Greenhalgh J. When trust, confidence, and faith collide: refining a realist theory of how and why inter-organisational collaborations in healthcare work. *BMC Health Services Research* 2021;**21**:602. https://doi.org/10.1186/s12913-021-06630-x

23. Goodwin N, 6 P, Peck E, Freeman T, Posaner R. *Managing across diverse networks of care: lessons from other sectors*: Health Services Management Centre, University of Birmingham; 2004.

24. Kumpunen S, Natasha Curry, Tim Ballard, Hannah Price, Mike Holmes, Nigel Edwards. *Collaboration in general practice: surveys of GPs and CCGs*. Slide pack. Nuffield Trust and Royal College of General Practitioners; 2015. https://www.nuffieldtrust.org.uk/research/collaboration-in-general-practice-surveys-of-gps-and-ccgs#partners.

25. Johl SK, Renganathan S. Strategies for gaining access in doing fieldwork: Reflection of two researchers. *Electronic Journal of Business Research Methods* 2010;**8**:42. https://academic-publishing.org/index.php/ejbrm/article/view/1251/1214.

26. Tubaro P, Ryan L, D'angelo A. The visual sociogram in qualitative and mixed-methods research. *Sociological Research Online* 2016;**21**:1-18. https://doi.org/10.5153/sro.3864.

27. Gale NK, Heath G, Cameron E, Rashid S, Redwood S. Using the framework method for the analysis of qualitative data in multi-disciplinary health research. *BMC medical research methodology* 2013;**13**:117. https://doi.org/10.1186/1471-2288-13-117.

For peer review only

28. QSR International Pty Ltd. (2018) NVivo (Version 12), https://www.qsrinternational.com/nvivo-qualitative-data-analysis-software/home.

29. NHS England and NHS Improvement. *Legislating for Integrated Care Systems: five recommendations to Government and Parliament*. London: NHS England and NHS Improvement; 2021. https://www.england.nhs.uk/wp-content/uploads/2021/02/legislating-for-integrated-care-systems-five-recommendations.pdf.

30. Parkinson S, Smith J. Primary care networks in a time of pandemic. *BJGP Life* 2020. https://bjgplife.com/primary-care-networks-in-a-time-of-pandemic/.

31. NHS England and NHS Improvemnt. *COVID-19 vaccination programme 2020/21 – next steps*; 2020. https://www.england.nhs.uk/coronavirus/wp-content/uploads/sites/52/2020/12/C0917-COVID-19-vaccination-programme-2020-21-next-steps-1-December-2020.pdf.

32. NHS England and NHS Improvement. *Update to GP contract arrangements for 2021/22*. London; 2021. https://www.england.nhs.uk/wp-content/uploads/2021/06/C1302-Update-to-GP-contract-arrangements-for-2021-22-.pdf.

33. NHS SDO. *Networks Briefing: Key lessons for network management in healthcare*. London: National Co-ordinating Centre for NHS Service Delivery and Organisation Research and Development; 2004.

34. Pettigrew LM, Kumpunen S, Rosen R, Posaner R, Mays N. Lessons for 'large-scale' general practice provider organisations in England from other inter-organisational healthcare collaborations. *Health Policy* 2019;**123**:51-61. https://doi.org/10.1016/j.healthpol.2018.10.017

35. Smith JA, Mays N. GP led commissioning: time for a cool appraisal. *BMJ* 2012;**344**:e980. https://doi.org/10.1136/bmj.e980.

36. Fisher R, Smith J, Sidhu M, Parkinson S, Alderwick H. *NHS reform: Five key questions about the future of primary care networks in England*. London: The Health Foundation; 2021. https://www.health.org.uk/publications/long-reads/nhs-reform.

37. Carter R, Riverin B, Levesque J-F, Gariepy G, Quesnel-Vallée A. The impact of primary care reform on health system performance in Canada: a systematic review. *BMC Health Services Research* 2016;**16**:324. https://doi.org/10.1186/s12913-016-1571-7

38. Taylor CJ, Wright M, Jackson CL, Hobbs R. Grass is greener? General practice in England and Australia. *The British journal of general practice : the journal of the Royal College of General Practitioners* 2016;**66**:428-9. https://doi.org/10.3399/bjgp16X686377

39. Thorlby R, Smith J, Barnett P, Mays N. Primary care for the 21st century: learning from New Zealands independent practitioner associations. Research report. 2012. https://www.nuffieldtrust.org.uk/files/2017-01/primary-care-21st-century-new-zealand-web-final.pdf.

For peer review only

FIGURE LEGEND

Figure 1: Sampling approach for selection of case studies

For peer review only

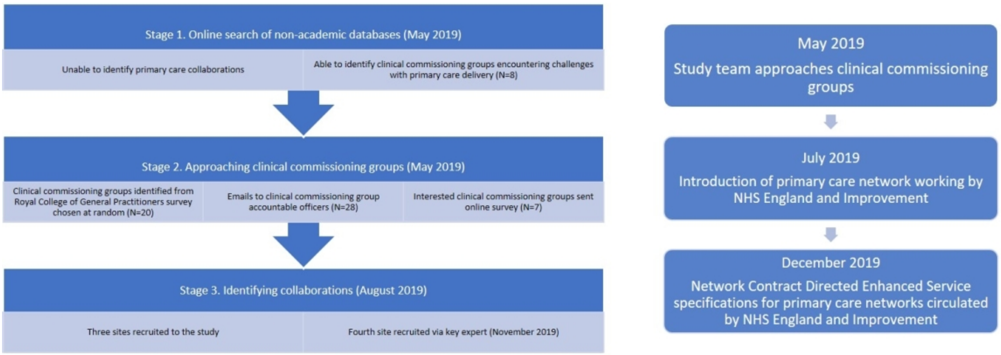

Figure 1: Summary of case study site sampling

253x89mm (150 x 150 DPI)

Research and reporting methodology
Revised **Standards for QUality Improvement Reporting Excellence** (**SQUIRE 2.0**) publication guidelines

Notes to authors

- The SQUIRE guidelines provide a framework for reporting new knowledge about how to improve healthcare.
- The SQUIRE guidelines are intended for reports that describe system level work to improve the quality, safety and value of healthcare, and used methods to establish that observed outcomes were due to the intervention(s).
- A range of approaches exists for improving healthcare. SQUIRE may be adapted for reporting any of these.
- Authors should consider every SQUIRE item, but it may be inappropriate or unnecessary to include every SQUIRE element in a particular manuscript.
- The SQUIRE glossary contains definitions of many of the key words in SQUIRE.
- The explanation and elaboration document provides specific examples of well-written SQUIRE items and an in-depth explanation of each item.
- Please cite SQUIRE when it is used to write a manuscript.

Text section and item name	Page/line no(s). info is located
**Title and abstract**	
1. **Title**	
Indicate that the manuscript concerns an initiative to improve healthcare (broadly defined to include the quality, safety, effectiveness, patient-centredness, timeliness, cost, efficiency and equity of healthcare).	Page 1
2. **Abstract**	
a. Provide adequate information to aid in searching and indexing.	Page 1
b. Summarise all key information from various sections of the text using the abstract format of the intended publication or a structured summary such as: background, local problem, methods, interventions, results, conclusions.	Page 1
**Introduction: Why did you start?**	
3. **Problem description** - Nature and significance of the local problem.	Pages 4-5
4. **Available knowledge** - Summary of what is currently known about the problem, including relevant previous studies.	Pages 4-5
5. **Rationale** - Informal or formal frameworks, models, concepts and/or theories used to explain the problem, any reasons or assumptions that were used to develop the intervention(s) and reasons why the intervention(s) was expected to work	Pages 4-5
6. **Specific aims** - Purpose of the project and of this report.	Page 6
**Methods: What did you do?**	
7. **Context** - Contextual elements considered important at the outset of introducing the intervention(s).	Pages 4-5

For peer review only

8. **Intervention(s)**	
a. Description of the intervention(s) in sufficient detail that others could reproduce it.	N/A – There is background on PCNs (key features in Box 1), but since this is a policy intervention it is not described in enough detail to reproduce it elsewhere
b. Specifics of the team involved in the work.	N/A – A brief description of each case study site is described in Table 2, but it was not possible to fully describe the teams involved in PCNs at a local level
9. **Study of the intervention(s)**	
a. Approach chosen for assessing the impact of the intervention(s).	Page 6
b. Approach used to establish whether the observed outcomes were due to the intervention(s).	N/A – Study was qualitative and did not focus on contribution/attribution towards outcome.
10. **Measures**	
a. Measures chosen for studying processes and outcomes of the intervention(s), including rationale for choosing them, their operational definitions and their validity and reliability.	Table 1 (Pages 6-7)
b. Description of the approach to the ongoing assessment of contextual elements that contributed to the success, failure, efficiency and cost.	Table 1 (Pages 6-7)
c. Methods employed for assessing completeness and accuracy of data.	Page 8
11. **Analysis**	
a. Qualitative and quantitative methods used to draw inferences from the data.	Pages 9-10
b. Methods for understanding variation within the data, including the effects of time as a variable.	N/A – Approach to analysing qualitative data considered variation between case study sites
12. **Ethical considerations** - Ethical aspects of implementing and studying the intervention(s) and how they were addressed, including, but not limited to, formal ethics review and potential conflict(s) of interest.	Page 2 (describes ethical approval)
**Results: What did you find?**	
13. **Results**	
a. Initial steps of the intervention(s) and their evolution over time (eg, time-line diagram, flow chart or table), including modifications made to the intervention during the project.	N/A – Policy intervention rather than intervention introduced by study team
b. Details of the process measures and outcomes.	Pages 10-13
c. Contextual elements that interacted with the intervention(s).	Pages 10-13

For peer review only

d. Observed associations between outcomes, interventions and relevant contextual elements.	Pages 10-13
e. Unintended consequences such as unexpected benefits, problems, failures or costs associated with the intervention(s).	Pages 10-13
f. Details about missing data.	Page 2 – Strengths and Limitations
**Discussion: What does it mean?**	
14. **Summary**	
a. Key findings, including relevance to the rationale and specific aims.	Page 13
b. Particular strengths of the project.	Page 2 – Strengths and Limitations<br>Page 15
15. **Interpretation**	
a. Nature of the association between the intervention(s) and the outcomes.	Page 13-15
b. Comparison of results with findings from other publications.	Page 13-15
c. Impact of the project on people and systems.	N/A – Paper discusses implications of research on management and oversight for PCNs, but does not describe impacts of project
d. Reasons for any differences between observed and anticipated outcomes, including the influence of context.	N/A – Nature of research questions discussed in paper do not rely on anticipated outcomes
e. Costs and strategic trade-offs, including opportunity costs.	N/A – Study considers complexity of PCNs, but does not specifically look at costs and trade-offs as an evaluation of a policy intervention
16. **Limitations**	
a. Limits to the generalisability of the work.	Page 2 – Strengths and Limitations
b. Factors that might have limited internal validity such as confounding, bias or imprecision in the design, methods, measurement or analysis.	Page 2 – Strengths and Limitations
c. Efforts made to minimise and adjust for limitations.	Page 2 – Strengths and Limitations
**Conclusions**	
a. Usefulness of the work.	Page 15
b. Sustainability.	Page 15
c. Potential for spread to other contexts.	Page 15
d. Implications for practice and for further study in the field.	Page 15
e. Suggested next steps.	Page 15

For peer review only

**Other information**	
18. **Funding** - Sources of funding that supported this work. Role, if any, of the funding organisation in the design, implementation, interpretation and reporting.	Page 2

Ogrinc G, et al. BMJ Qual Saf 2015;0:1–7. doi:10.1136/bmjqs-2015-004411
Downloaded from http://qualitysafety.bmj.com/ on January 2, 2017

For peer review only
